# De Novo *DNM1L* Mutation in a Patient with Encephalopathy, Cardiomyopathy and Fatal Non-Epileptic Paroxysmal Refractory Vomiting

**DOI:** 10.3390/ijms25147782

**Published:** 2024-07-16

**Authors:** Beatrice Berti, Daniela Verrigni, Alessia Nasca, Michela Di Nottia, Daniela Leone, Alessandra Torraco, Teresa Rizza, Emanuele Bellacchio, Andrea Legati, Concetta Palermo, Silvia Marchet, Costanza Lamperti, Antonio Novelli, Eugenio Maria Mercuri, Enrico Silvio Bertini, Marika Pane, Daniele Ghezzi, Rosalba Carrozzo

**Affiliations:** 1 Centro Clinico Nemo and Pediatric Neurology Unit, Fondazione Policlinico Universitario A. Gemelli IRCCS, Largo Agostino Gemelli, 8, 00168 Rome, Italy; beatrice.berti@policlinicogemelli.it (B.B.); daniela.leone@policlinicogemelli.it (D.L.); concetta.palermo@policlinicogemelli.it (C.P.); eugeniomaria.mercuri@unicatt.it (E.M.M.); marika.pane@policlinicogemelli.it (M.P.); 2 Translational Cytogenomics Research Unit, Bambino Gesù Children’s Hospital, IRCCS, 00165 Rome, Italy; daniela.verrigni@opbg.net (D.V.); antonio.novelli@opbg.net (A.N.); 3 Unit of Medical Genetics and Neurogenetics, Fondazione IRCCS Istituto Neurologico Carlo Besta, 20126 Milan, Italy; alessia.nasca@istituto-besta.it (A.N.); andrea.legati@istituto-besta.it (A.L.); silvia.marchet@istituto-besta.it (S.M.); costanza.lamperti@istituto-besta.it (C.L.); daniele.ghezzi@istituto-besta.it (D.G.); 4 Unit of Cell Biology and Diagnosis of Mitochondrial Disorders, Laboratory of Medical Genetics, Bambino Gesù Children’s Hospital IRCCS, 00146 Rome, Italy; dinottia.michela@opbg.net (M.D.N.); alessandra.torraco@opbg.net (A.T.); teresa.rizza@opbg.net (T.R.); 5 Neuromuscular Disorders Research Unit, Bambino Gesù Children’s Hospital IRCCS, 00165 Rome, Italy; enricosilvio.bertini@opbg.net; 6 Molecular Genetics and Functional Genomics, Bambino Gesù Children’s Hospital, IRCCS, 00165 Rome, Italy; emanuele.bellacchio@opbg.net; 7 Pediatric Neurology Unit, Università Cattolica del Sacro Cuore, Largo Francesco Vito, 1, 00168 Rome, Italy; 8 Department of Pathophysiology and Transplantation, University of Milan, 20122 Milan, Italy

**Keywords:** *DNM1L*, cardiomyopathy, mitochondrial disorders, paroxysmal vomiting, mitochondrial dynamics, mitochondrial fission

## Abstract

Mitochondrial fission and fusion are vital dynamic processes for mitochondrial quality control and for the maintenance of cellular respiration; they also play an important role in the formation and maintenance of cells with high energy demand including cardiomyocytes and neurons. The *DNM1L* (dynamin-1 like) gene encodes for the DRP1 protein, an evolutionary conserved member of the dynamin family that is responsible for the fission of mitochondria; it is ubiquitous but highly expressed in the developing neonatal heart. De novo heterozygous pathogenic variants in the *DNM1L* gene have been previously reported to be associated with neonatal or infantile-onset encephalopathy characterized by hypotonia, developmental delay and refractory epilepsy. However, cardiac involvement has been previously reported only in one case. Next-Generation Sequencing (NGS) was used to genetically assess a baby girl characterized by developmental delay with spastic–dystonic, tetraparesis and hypertrophic cardiomyopathy of the left ventricle. Histochemical analysis and spectrophotometric determination of electron transport chain were performed to characterize the muscle biopsy; moreover, the morphology of mitochondria and peroxisomes was evaluated in cultured fibroblasts as well. Herein, we expand the phenotype of *DNM1L*-related disorder, describing the case of a girl with a heterozygous mutation in *DNM1L* and affected by progressive infantile encephalopathy, with cardiomyopathy and fatal paroxysmal vomiting correlated with bulbar transitory abnormal T2 hyperintensities and diffusion-weighted imaging (DWI) restriction areas, but without epilepsy. In patients with *DNM1L* mutations, careful evaluation for cardiac involvement is recommended.

## 1. Introduction

Mitochondria are highly dynamic organelles that undergo fusion, fission and mitophagy. These processes are essential to maintain normal mitochondrial morphology, distribution, and function [1,2]. Disorders of mitochondrial dynamics are caused by pathogenic variants in the genes encoding proteins involved in these processes; they are inherited in an autosomal recessive or dominant manner and include a group of various diseases that range in severity from isolated optic atrophy to lethal encephalopathy [3,4].

Mitochondrial fission is necessary to generate new mitochondria prior to cell division, allowing the transport and redistribution of mitochondria inside the cell and facilitating the segregation of damaged organelles for mitophagy. Inversely, mitochondrial fusion enables the exchange of intra-mitochondrial material between mitochondria [2].

The *DNM1L* (dynamin-1 like) gene encodes for the DRP1 protein, an evolutionary conserved member of the dynamin family, responsible for the fission of mitochondria and having a role in the maintenance of mitochondrial and peroxisomal morphology. DRP1 impairment is implicated in several neurological disorders associated with either dominant or recessive *DMN1L* mutations (MIM #603850) and presenting with two main distinct phenotypes. Inherited heterozygous *DNM1L* pathogenic variants can cause an autosomal dominant form of isolated optic atrophy [5]. Conversely, a more severe disease, *DNM1L*-related encephalopathy, can be caused by either monoallelic de novo dominant or biallelic loss-of-function variants in this gene [6,7,8]. Mutations that fall into the GTPase domain are typically associated with a recessive trait (except for the cases not associated with early encephalopathy [5]), while mutations that fall into the middle domain are expressed as dominant-negative alleles [9].

De novo heterozygous pathogenic variants in *DNM1L* have been previously reported to be associated with neonatal or infantile-onset encephalopathy characterized by hypotonia, developmental delay and refractory epilepsy. Brain Magnetic Resonance Imaging (MRI) generally shows progressive cerebral atrophy and transitory abnormal T2 hyperintensities and diffusion tensor imaging (DTI) restriction areas in basal ganglia or cortical areas that are quite characteristic for *DNM1L* encephalopathy [9].

Epilepsy is a common feature in mitochondrial dynamics disorders, often part of a multisystem clinical presentation; it may be very difficult to manage and has a poor outcome in many cases. Multiple antiepileptic drugs (AEDs) are frequently needed to achieve control of seizures that become refractory to treatment in the terminal stages of the disease [10,11]. Previous cases reported a frequent association between *DNM1L* mutations and encephalopathy with recurrent status epilepticus (focal or generalized) followed by cognitive regression and death in early childhood [7,12,13].

Cardiac involvement is quite a common feature in mitochondrial disorders. However, cardiomyopathy seems rarely present in *DNM1L*-related phenotypes, and only one recent report describes that cardiac involvement can be a clinically important feature of *DNM1L*-related disorders [14].

Decreased activity of electron transport chain (ETC) complexes in muscle and fibroblasts have been seldom observed; in fact, most patients do not have biochemical evidence of mitochondrial or peroxisomal dysfunction. However, microscopy analyses in cultured fibroblasts typically show elongated, tangled and hyperfused mitochondria and peroxisomes [9,12].

Herein, we expand the phenotype of *DNM1L*-related disorder describing the case of a girl with a reported mutation in *DNM1L* [15] and affected by progressive infantile encephalopathy, with cardiomyopathy and fatal paroxysmal vomiting correlated to bulbar transitory abnormal T2 hyperintensities and DWI restriction areas but without epilepsy.

## 2. Results

### 2.1. Clinical Characteristics

The proband was born to healthy, non-consanguineous Caucasian parents following normal pregnancy. Delivery was normal at term, and there was no concern in the first months of life. The family sought medical attention at 7 months of age because she presented strabismus and motor regression (drop of head control). Subsequently, she developed a severe global developmental delay with spastic–dystonic tetraparesis with minor cognitive impairment. Several clinical, genetic and metabolic investigations were undertaken. Electroneurography disclosed a moderate–severe axonal motor polyneuropathy. Serial brain MRI (performed at 12, 24, 36 months of age) was normal. Comparative Genomic Hybridization (CGH)-array and gene testing for mutations in *PLA2G6*, *PANK2* and *SPG7* were negative, as well as NGS-based panel screening of 102 genes associated with movement disorders in childhood. Due to the progressive evolution of neuromuscular involvement in the absence of any metabolic alteration at blood tests (serum lactate, pyruvate and alanine), muscle and skin biopsies were performed at 3 years of age. Histochemical analysis did not reveal any deficiency of Cytochrome c Oxidase (COX) and Succinate DeHydrogenease (SDH) reactivity in muscle (Supplementary Figure S1a), and spectrophotometric determination of ETC showed normal activities (Supplementary Figure S1b).

At the age of 6 years, she started to present recurrent episodes of vomiting, remitting spontaneously after 3–4 days, and was unresponsive to various treatments (ondansetron, metoclopramide, cyproheptadine, domperidone, and Proton Pump Inhibitors). The frequency of these episodes ranged from monthly to every half-year. Serial esophagogastroduodenoscopy and abdomen ultrasound were performed, all resulting negative. Due to poor growth, a gastrostomy feeding tube was placed at 9 years of age with a good outcome but with persistence of paroxysmal vomiting.

**Figure 1:**
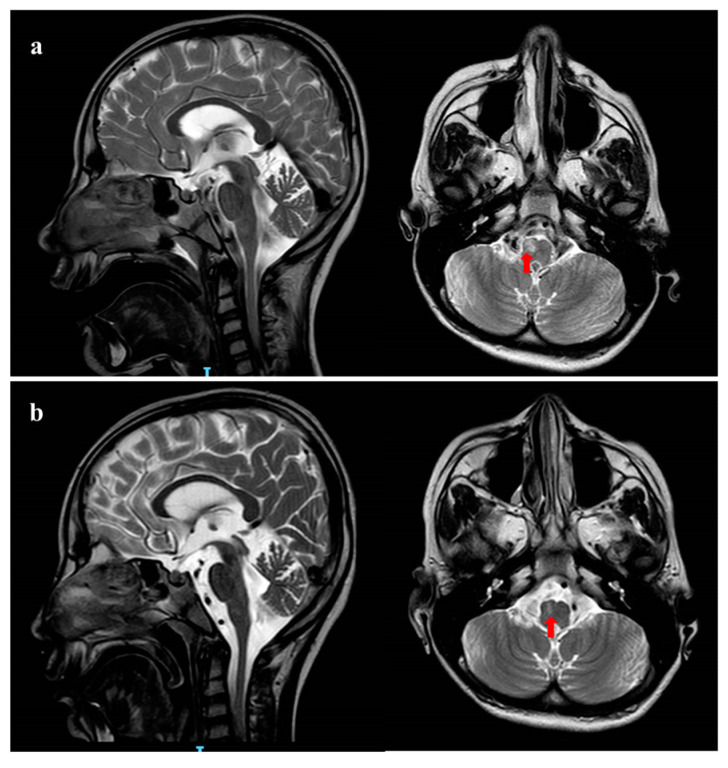
Brain MRI pattern. (**a**) Brain MRI performed in the acute phase at 10 years of age. Coronal and Sagittal T2 weighted images shows global cerebral and cerebellar atrophy. Red arrow shows hyperintensity of the right bulbar pyramid; (**b**) brain MRI performed 20 days later shows reduction in the right corresponding bulbar pyramid lesion.

Due to the worsening of gastric symptoms, she was hospitalized at the age of 10 years. Brain MRI performed in acute phase revealed moderate global cerebral and cerebellar atrophy and T2-FLAIR (Flow-attenuated inversion recovery) hyperintensity of right bulbar pyramid with DWI restriction (Figure 1a), while MRI spectroscopy did not detect any lactate peak.

**Figure 2:**
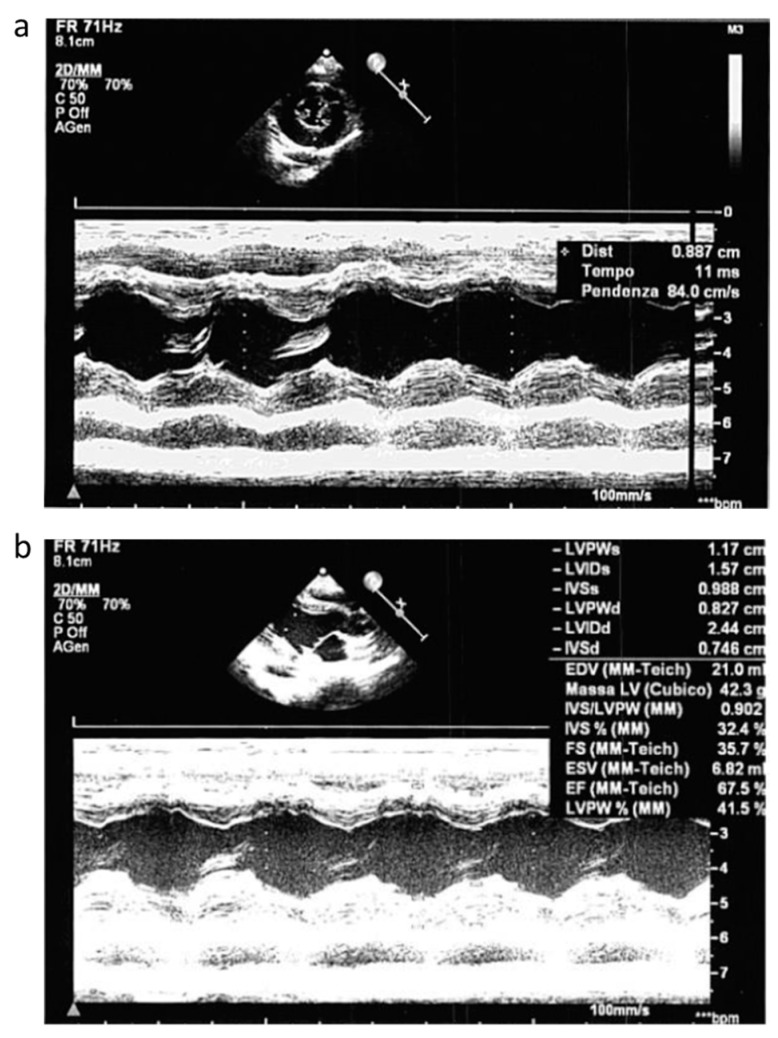
Echocardiogram pattern. Echocardiogram performed during acute phase that showed moderate hypertrofic cardiomyopathy of the left ventricle (**a**) without outflow obstruction and with normal EF (**b**).

Concomitant cardiac evaluation with echocardiogram showed a moderate hypertrophic cardiomyopathy of the left ventricle (Figure 2a) without outflow obstruction and with normal ejection fraction (EF) (Figure 2b).

During the follow-up, a worsening of the clinical picture occurred with refractory vomiting, respiratory failure and progressive neurologic degradation (GCS < 5). Prolonged EEG showed slowing background without a clear epileptiform activity; several AED therapies (continuous intravenous midazolam, levetiracetam, clonazepam) did not change either the clinical picture nor the electroencephalography (EEG) pattern. Blood tests for mitochondrial disorders (serum lactate and pyruvate) remained normal. A control brain MRI performed 20 days later showed moderate reduction in the right bulbar pyramid lesion without DWI restriction, suggesting ischemic origin (Figure 1b). After two months of hospitalization, the patient died from multisystem failure.

### 2.2. Genetic and Structural Analysis

**Figure 3:**
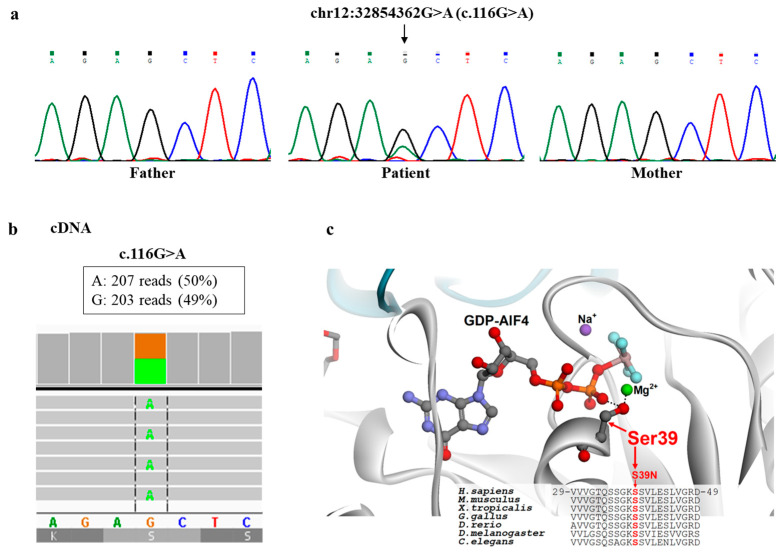
Genetic and structural analysis. (**a**) The c.116G>A variant identified by NGS in *DNM1L* was confirmed by Sanger sequencing in the patient and appeared absent in both parents; (**b**) cDNA, obtained by retrotranscription of mRNA from patient’s fibroblasts, revealed balanced expression of the two alleles; (**c**) DRP1 structure and multiple protein sequence alignment around the site of the p.Ser39Asn replacement. Crystal structure of the human dynamin-1-like protein in complex with GDP-AlF4 (Protein Data Bank code 3W6P) and protein sequence alignment around Ser39, highlighting the role of this serine in the binding of the GTP/GDP ligand and its conservation among different organisms.

Using the clinical exome, we did not find any biallelic variant compatible with a recessive inheritance, whereas using a de novo dominant model, we identified a heterozygous variant (NM_001278464.2: c.116G>A, p.Ser39Asn) in *DNM1L*, predicted pathogenic (PM1, PM5, PP3, PP5, PM2, PM6), that was detected in the proband but absent in parents (Figure 3a). *DNM1L* transcript analysis of cDNA, obtained by retrotranscription of mRNA from fibroblasts, revealed balanced expression of the two alleles (Figure 3b) and did not show any alternative transcript. This finding indicated that the identified c.116G>A variant was not associated with nonsense mRNA decay or aberrant splicing and ruled out the possibility of a second *DNM1L* mutation that was not detected by NGS.

The p.Ser39Asn variant hits an invariant residue in the binding site of GTP/GDP that interacts with the phosphate moiety of the cofactor (Figure 3c). The replacement of Ser39 with an asparagine residue is expected to impair the interactions of DRP1 with GTP/GDP, thus impairing the GTP hydrolysis-dependent oligomerization of the protein, which has a crucial function in the dynamics of mitochondrial and peroxisomal membranes.

### 2.3. Biochemical Analysis, Immunostaining and Imaging in Fibroblasts

**Figure 4:**
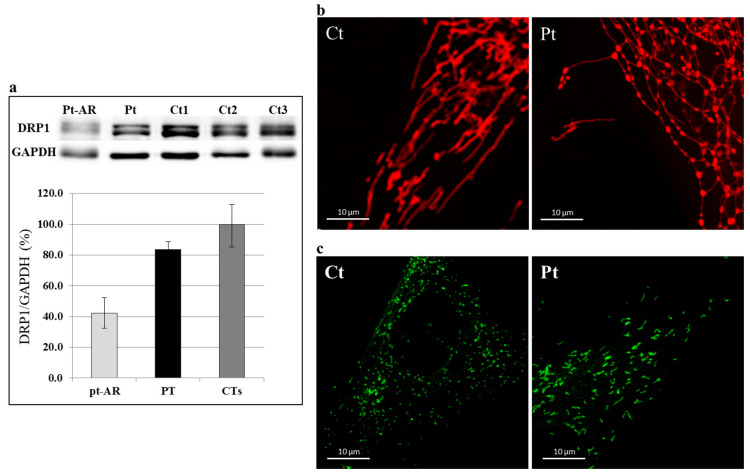
Western blotting and immunostaining analysis. (**a**) Immunoblot analysis of total lysates from controls (Ct) and patient (Pt) fibroblasts using DRP1 and GAPDH antibodies. The latter was used as loading control. The steady-state level of DRP1 protein in the patient is in the low range of normal controls. Fibroblasts from a patient with recessive *DNM1L* mutations (Pt-AR) were used as “positive” control. Values in the graph are given as the mean ± SD (n = 4–5). PT vs. CTs: *p*-value = 0.018. (**b**) Characterization of the mitochondrial network: representative images of mitochondrial morphology in control (Ct) and patient (Pt) fibroblasts grown in galactose-supplemented medium. Mitochondrial network of *DNM1L*-mutant fibroblasts showed an altered mitochondria morphology, with swollen, dots, and “chain-like” structures. (**c**) Characterization of the peroxisomal network: immunofluorescence staining with the anti-PMP70 antibody of fibroblasts from controls (Ct) and patient (Pt). Fibroblasts from patient displayed organelles longer and larger compared with control. (Scale bar = 10 μm).

In order to prove the deleterious effect of the identified *DNM1L* variant, we analyzed fibroblasts from the patient. First, we investigated the DRP1 protein by immunoblot analysis; in the patient’s cells, the amount of DRP1 was similar to controls (Figure 4a) in contrast to a subject with biallelic *DNM1L* mutations who displayed a clearly reduced amount. Next, we evaluated the mitochondrial network by Mitotracker red staining. We used galactose-supplemented medium, a stressing condition that forces cells to use the oxidative phosphorylation for ATP production; in this condition, the mitochondria of control cells tend to elongate and fuse, while the mitochondrial network of *DNM1L*-mutant fibroblasts differ from the behavior of controls mitochondria in stress conditions, displaying morphologically altered mitochondria, with swollen, dots, and “chain-like” structures (Figure 4b and Figure S2). Given that DRP1 also contributes to the division of peroxisomes, we evaluated the morphology of these organelles by using an antibody against a peroxisomal protein, PMP70. In *DNM1L*-mutant cells, we observed organelles to be larger and more elongated, in contrast with the dotted staining observed in control cells (Figure 4c and Figure S3).

## 3. Discussion

*DNM1L*-related disorders are associated with neonatal or infantile-onset encephalopathy with varying severity degrees of refractory epilepsy, hypotonia and cognitive impairment dependent on the specific causative variant. Herein, we further expand the clinical spectrum of *DNM1L*-associated mutations reporting the case of a de novo heterozygous variant (c.116G>A, p.Ser39Asn), recently described [15], and associated in our patient with progressive infantile encephalopathy, cardiomyopathy and paroxysmal refractory vomiting, probably due to subcortical involvement, but without epilepsy. Unusual clinical findings in our case were the heart’s involvement and the recurrent progressive paroxysmal vomiting.

The heart is the main organ with the greatest energy demand, and it relies heavily on mitochondria to obtain adequate energy for its functions. Notably, it has been observed that DRP1 downregulation hampers the clearance of mitochondria by autophagy, causing mitochondrial dysfunction under basal and stressed conditions, thus playing a central role in maintaining cardiac homeostasis. Indeed, quality-control mechanisms in cardiomyocytes, involving mitochondrial biogenesis, dynamics and mitophagy, protect them against mitochondrial dysfunction [16,17]. The overall importance of DRP1 functions is underlined by the finding that Drp1 null mice approximately died within ten days of embryonic life, showing elongated mitochondria, reduced cell proliferation and decreased developmentally regulated apoptosis [18]. Moreover, there is some evidence of the relationship between DRP1 dysregulation and cardiac defects. It has been reported that Drp1KO cardiomyocytes displayed increased mitochondria connectivity, accumulation of ubiquitinated proteins, and decreased respiration, leading to cardiomyopathy and rapid lethality in mouse models [16,17,19,20]. In addition, it has been shown that the dynamics of mtDNA nucleoids regulated by mitochondrial fission is crucial for neonatal cardiomyocyte development by promoting homogeneous distribution of active mitochondria throughout the cardiomyocytes [21,22]. Indeed, DRP1 is highly expressed in the developing neonatal heart. Notably, muscle-specific Drp1 knockout (Drp1-KO) mice showed neonatal lethality due to dilated cardiomyopathy [21], and other studies reported association between *DNM1L* and cardiac involvement in mouse models [22]. A single other recent paper described cardiac involvement, leading to congestive heart failure and death, in a subject with a dominant de novo *DNM1L* mutation [14].

Moreover, our patient presented episodes of paroxysmal vomiting, not associated with epileptic abnormalities at ictal EEG, and appeared to be correlated to the transitory brain stem lesion documented by the MRI abnormalities of the bulbar pyramidal tract.

The variant is predicted to cause the amino acid change p.Ser39Asn (NP 036192), affecting a highly conserved residue and with high scores of pathogenicity [23], according to different bioinformatics tools (Polyphen2, SIFT, Mutation Taster). In particular, Ser39 lies in the binding pocket for GTP and, in analogy with previously reported amino acid substitutions in the same region, it impairs the interactions of DRP1 with GTP [7] and the GTP/GDP hydrolysis-dependent structural dynamics of this protein.

In conclusion, we have confirmed that cardiac involvement, previously reported only in one patient and in the mouse Drp1-KO model, may occur, although it is a rare finding in *DNM1L*-related encephalopathy. Furthermore, an additional clinically important feature associated with a *DNM1L* mutation in our patients is fatal non-epileptic paroxysmal refractory vomiting, likely related to a brain stem bulbar’s involvement.

## 4. Materials and Methods

### 4.1. Histochemistry and Biochemistry

Cryostatic cross-sections of the muscle biopsy were processed according to standard histochemical procedures. ETC complex activities were measured using standard spectrophotometric methods [24] in muscle homogenate and digitonin-treated skin fibroblasts. Values were normalized to citrate synthase (CS).

### 4.2. Genetic Studies

Total DNA from patient and parents was isolated using the QIAamp DNA Mini Kit (Qiagen, Valencia, CA). DNA was analyzed using the TruSightOneExpanded panel (clinical exome, Illumina, San Diego, CA, USA), comprehensive of greater than 6.700 clinically relevant genes, and underwent high-throughput sequencing on a NextSeq 500 System (Illumina, San Diego, CA, USA). Prioritization of the variants was achieved by selecting variants having allele frequency <0.01 according to public database gnomAD, https://gnomad.broadinstitute.org/ (accessed on 1 January 2024); or our in-house database, and considering quality scores (e.g., coverage > 20), amino acid impact (high or moderate according to in silico prediction tools), and possible clinical significance. Sanger sequencing analysis, using BigDye chemistry 3.1 and run on an ABI 3130XL automatic sequencer (Applied Biosystems, Life Technologies Italia, Segrate, Italy), was performed to confirm the presence of the identified variants in the proband and to follow the segregation analysis within the parents.

### 4.3. cDNA Analysis

For RNA purification from fibroblasts and cDNA retrotranscription, we used RNeasy mini kit (QIAGEN) and GoTaq 2-Step RT-qPCR System (Promega), respectively, according to the manufacturers’ protocols. For sequencing of *DNM1L* transcript, PCR products were processed with Nextera XT DNA sample preparation kit (Illumina). Next-generation sequencing (NGS) was performed on an Illumina MiSeq instrument.

### 4.4. Immunoblot

A total of 50 μg of protein from fibroblast pellets was loaded for each sample in denaturing SDS polyacrylamide gel electrophoresis [7]. A polyclonal antibody against DRP1 (D6C7, #8570, Cell Signaling; dilution 1:1000) and a monoclonal antibody against GAPDH (#MAB374, Millipore; dilution 1:1000), were used. Data obtained by densitometry analysis were calculated as the mean of replicates ± standard deviation (SD) and then compared with an unpaired two-tailed *t*-test.

### 4.5. Fluorescence Microscopy

For visualization of the mitochondrial network, the mitochondrial fluorescent dye MitoTracker Red-CMXRos (Invitrogen, Waltham, MA, USA) was added to the culture media at a final concentration of 50 nM for 30 min and then visualized by fluorescence microscopy [7]. For visualization of peroxisomes, after fixation and permeabilization, cells were incubated with anti-PMP70 (ABT12, Millipore; dilution 1:200) antibody, followed by a fluorescently labeled secondary antibody (Alexa Fluor 488, Invitrogen; dilution 1:1000). Images were acquired with a confocal microscope (Leica TSC-SP8).

## Data Availability

The data supporting the findings of this study are available within this article. Raw data of the fluorescence microscopy experiments are available on the repository Zenodo (10.5281/zenodo.12582727).

## Supplementary Materials

The supporting information can be downloaded at: https://www.mdpi.com/article/10.3390/ijms25147782/s1.
