# Supplementary material for: De Novo DNM1L Mutation in a Patient with Encephalopathy, Cardiomyopathy and Fatal Non-Epileptic Paroxysmal Refractory Vomiting
Source: Int J Mol Sci. 2024 Jul 16;25(14):7782. doi: 10.3390/ijms25147782 (PMC11277250; doi:10.3390/ijms25147782)
Supplement: Supplementary file 1 [file ijms-25-07782-s001.zip › ijms-3058147-supplementary.pdf]

a

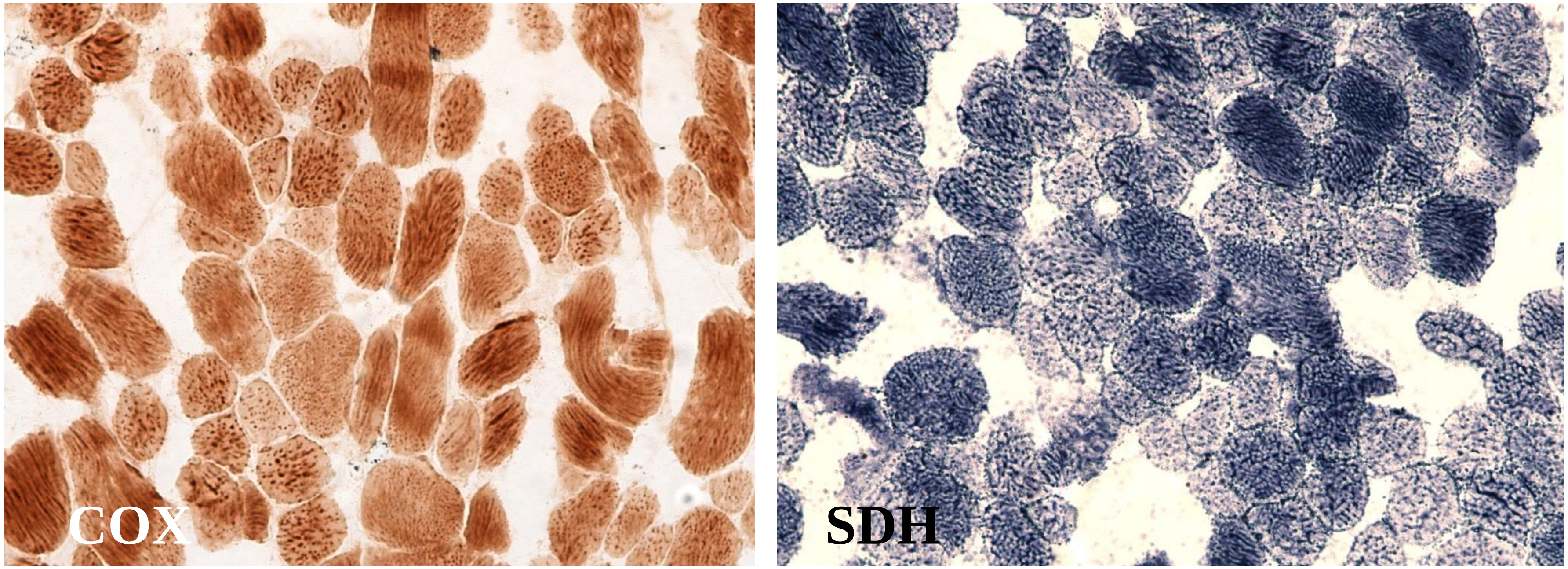

b

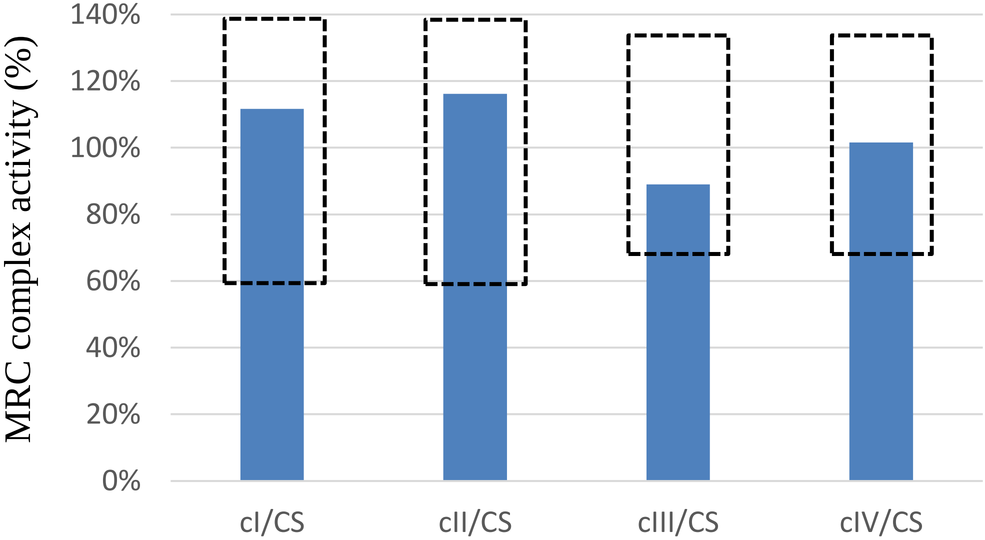

Supplementary Figure S1: Histochemical and biochemical analyses

a) Images of the cytochrome c oxidase (COX) and succinate dehydrogenase (SDH) stainings performed on muscle sample from the proband.

b) Biochemical activity of mitochondrial respiratory chain (MRC) complexes in muscle. All enzymatic activities are normalized for Citrate Synthase (CS) activity and reported as percentage of the control mean. Dashed boxes correspond to the control range.

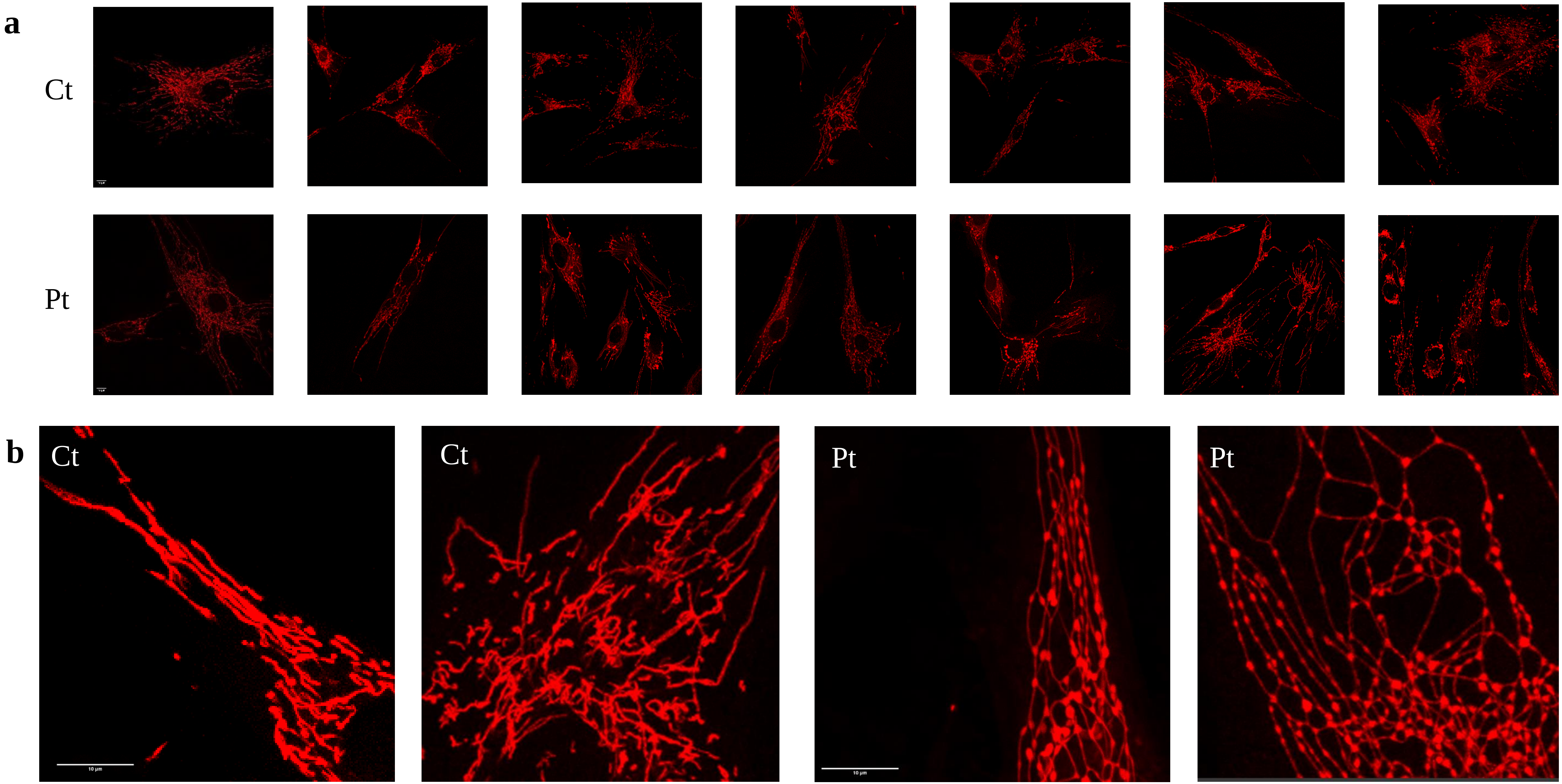

Supplementary Figure S2: Characterization of the mitochondrial network

a) Series of images of mitochondrial morphology staining by Mitotracker red in control (Ct) and patient (Pt) fibroblasts grown in galactose-supplemented medium.

b) Digital zoom 4x of the mitochondrial network staining. Scale bars: 10µm.

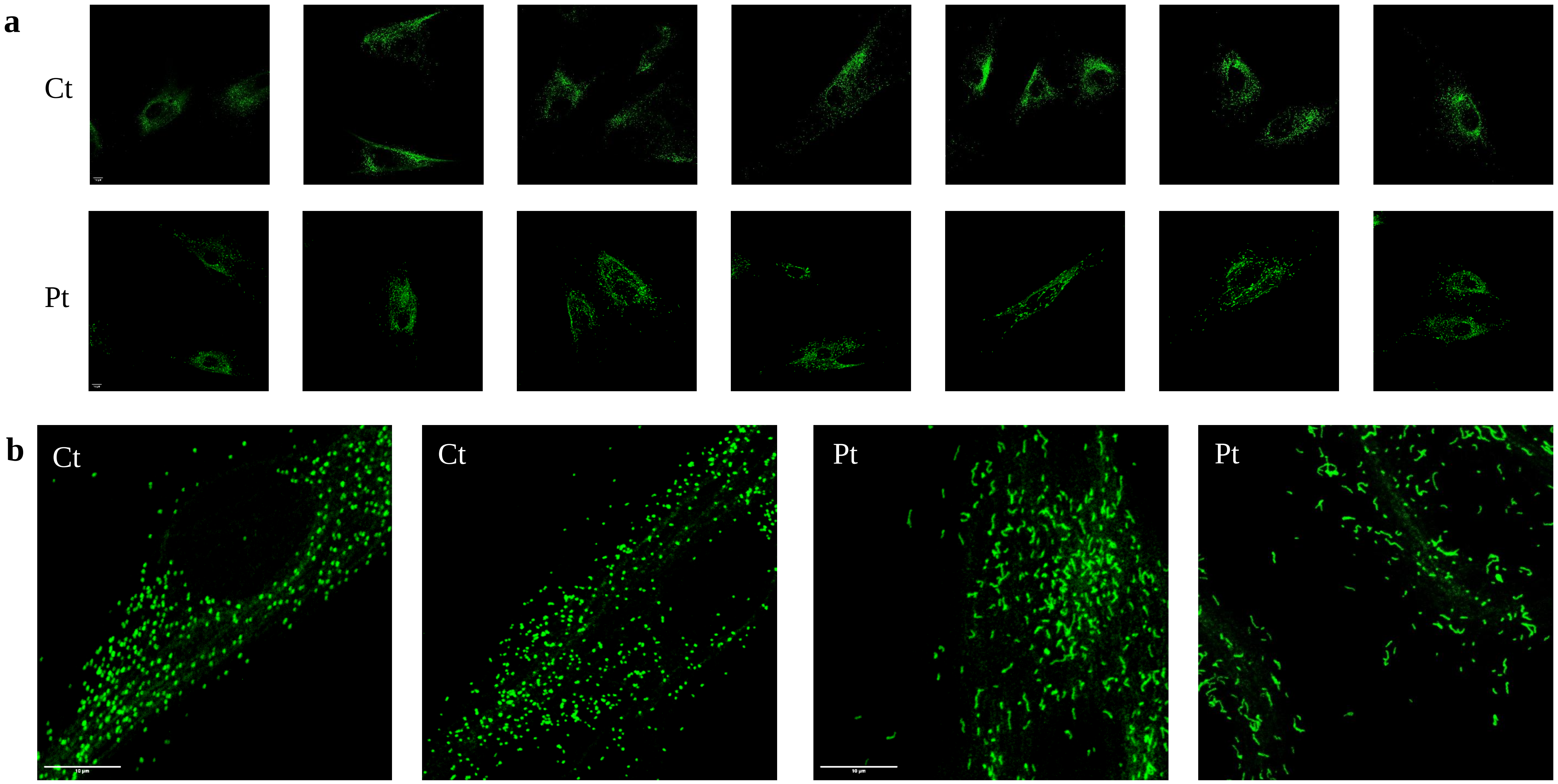

Supplementary Figure S3: Characterization of the peroxisomal morphology

a) Series of images of the peroxisomes obtained by immunofluorescence staining with the anti-PMP70 antibody of fibroblasts from controls (Ct) and patient (Pt).

b) Digital zoom 4x of the peroxisomal morphology staining. Scale bars: 10µm.
